# Switching to Fully Online Teaching and Learning of Mathematics: The Case of Norwegian Mathematics Lecturers and University Students During the Covid-19 Pandemic

**DOI:** 10.1007/s40753-021-00162-9

**Published:** 2022-01-18

**Authors:** Farzad Radmehr, Simon Goodchild

**Affiliations:** 1 Norwegian University of Science and Technology, Trondheim, Norway; 2 University of Agder, Kristiansand, Norway; 3 Western Norway University of Applied Sciences, Bergen, Norway

**Keywords:** Pedagogical issues, Post-secondary education, Teacher professional development

## Abstract

Towards the end of 2019, a novel coronavirus, known as COVID-19, was detected and quickly spread worldwide. The resulting pandemic led many countries to lockdown and teaching and learning switched to fully online provision. This study explores how Norwegian higher education lecturers and students of mathematics experienced online provision following this switch in March 2020 when the national lockdown was imposed. Data are generated and analysed using an exploratory sequential mixed methods approach that first entailed interviews with ten mathematics lecturers and six undergraduate students as the foundation for developing a survey instrument. The instrument was designed to explore further how a larger sample of mathematics lecturers and students perceived their experiences following the switch to online teaching and learning. One hundred and twenty-seven university students from four universities and eighteen mathematics lecturers from seven universities responded to the survey. The data generated indicate that advanced technology and the internet were not entirely successful in supporting many students and lecturers to adjust to the lockdown environment. Additionally, it appears that some mathematics lecturers were not aware of several challenges that students experienced following the switch. This paper aims to increase the awareness of the mathematics education community at the tertiary level about the challenges mathematics lecturers and students experience through online education. Further, it is hoped to prompt collaboration within the community to address these challenges in order to be better equipped for any use of online teaching and learning of mathematics in higher education.

## Introduction

We report from a survey of mathematics lecturers and students working in higher education in Norway that was designed to explore their experiences of teaching and learning following a mandated switch to online education. The switch in March 2020 was precipitated by the COVID-19 pandemic that emerged at the beginning of the year. It resulted in many countries enforcing a lockdown to reduce the exponential growth rate, consequent overload of health services, and loss of life^1^.

We claim that Norway is a special case and thus of interest for several reasons. As an advanced and wealthy country, the use of technology in education was reasonably well-developed with widespread ready access to computers and mobile technology. Moreover, probably due to the dispersion of a relatively small population (circa 5.5 million) over a country that extends over 24° latitude (circa 2,500 km), broadband (fibre and mobile) connections are well-developed and widely used by the population at large. Moreover, there is comprehensive local institutional provision for online library and IT services and the use of learning management software, in addition to national software (licence) agreements, such as for video conferencing and administrative systems. Nevertheless, the switch to online teaching and learning came rather suddenly (with just three days warning – including a weekend) and many mathematics teachers were rather ill-prepared despite the well-developed infrastructure.

The use of digital technologies in education has been the focus of research for several decades, and some advanced software solutions for the challenges of teaching and learning have been developed, especially in the context of STEM subjects (e.g., Borba et al., 2017; Engelbrecht et al., 2020; Maciejewski, 2016; Naccarato & Karakok, 2015). This research and development form the backdrop for the inquiry we report. Thus, we report from a context with a highly developed and accessible digital infrastructure for supporting the switch to online teaching and learning mathematics in higher education. But, we report on a population of teachers that had, to a large extent, allowed the structural developments to take place without affecting their practice to any great extent. In this context, we address the following research question: *What was the experience of Norwegian university mathematics lecturers and students following the sudden switch to fully online teaching imposed by the national lockdown enforced in reaction to the COVID-19 pandemic in 2020?*

## Theoretical Framework

**Fig. 1:**
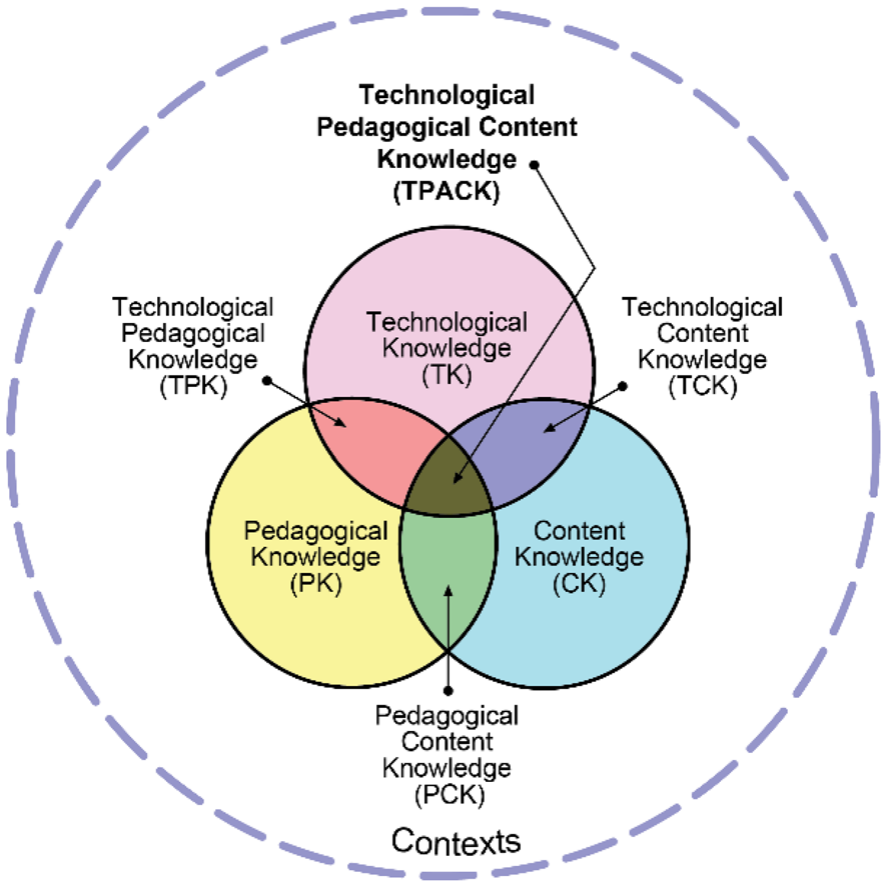
*The TPACK framework*. Adopted from Koehler and Mishra (2009, p. 63)

This study is framed within the theory of Technological Pedagogical Content Knowledge (TPACK) (Fig. 1) (Koehler & Mishra, 2009; Mishra & Koehler, 2006). This framework was developed over a five-year design experiment focusing on how teachers and lecturers at schools and universities could develop their teaching with technology (Mishra & Koehler, 2006).

TPACK highlights the importance of understanding how technology could be used for effective teaching in a digital environment in teachers’ knowledge. Specifically, this framework points to three core knowledge types (i.e., content, pedagogical, and technological) at the centre of effective teaching with technology (Mishra & Koehler, 2008). These three knowledge types are interconnected, and the relationships between them are as important as the individual types (Mishra & Koehler, 2008). Social and contextual factors are highlighted as a significant dimension of TPACK because the three knowledge types can interact differently across a variety of contexts and differentially impact teachers’ decisions on integrating technology in teaching (Koehler & Mishra, 2009; Mishra & Koehler, 2008). This framework has been used frequently in studies related to integrating technology into teaching both broadly in educational research (e.g., Archambault & Barnett, 2010; Yeh et al., 2021) and also in mathematics education studies (e.g., Lee & Hollebrands, 2008; Zambak & Tyminski, 2020).

TPACK, introduced by Mishra and Koehler (2006), is a development of Shulman's (1986) construct of pedagogical content knowledge (PCK). As illustrated in Fig. 1, the TPACK framework has seven components. Three components are those defined by Shulman (1986) (i.e., content knowledge (CK), pedagogical knowledge (PK), and PCK) and four introduced by Mishra and Koehler (2006) to accommodate the integration of technology into teaching (i.e., technological knowledge (TK), technological content knowledge (TCK), technological pedagogical knowledge (TPK), and technological pedagogical content knowledge (TPACK)). These seven components are well described in Koehler and Mishra (2009) and Mishra and Koehler (2006). Here we briefly describe the components related to technology, that is TK, TCK, TPK, and TPACK. TK is always under development and it is beyond the traditional definition of computer literacy; it includes understanding how and when information technology could help or hinder achieving a goal (Koehler & Mishra, 2009). TCK could be defined as “an understanding of the manner in which technology and content influence and constrain one another” (Koehler & Mishra, 2009, p. 65). TPK is about how different technologies can impact teaching and learning, including their pedagogical affordances and constraints. Teachers with high TPK are creatively exploring how different technologies could be used to improve students’ learning (Koehler & Mishra, 2009). Finally, TPACK includes.

…an understanding of the representation of concepts using technologies; pedagogical techniques that use technologies in constructive ways to teach content; knowledge of what makes concepts difficult or easy to learn and how technology can help redress some of the problems that students face; knowledge of students’ prior knowledge and theories of epistemology; and knowledge of how technologies can be used to build on existing knowledge and to develop new epistemologies or strengthen old ones (Mishra & Koehler, 2006, p. 1029).

Teachers’ development of TPACK is critical for teaching effectively with technology (Koehler & Mishra, 2009). To improve the quality of teaching, TPACK should be applied in all (digitally resourced) teaching situations, and teachers should be aware that no single technological solution works for all situations (Koehler & Mishra, 2009).

Previous research on TPACK as a framework focused more on teacher education and teachers at kindergarten and schools. A few studies have explored TPACK at the tertiary level (Fabian et al., 2019). Benson and Ward (2013), through conducting a qualitative case study with three faculty members, found that high TK alone is not enough for developing TPACK, and a high level of PK is the enabling force in the development of this knowledge. However, this does not mean TK is not important when focusing on TPACK development. TK should not be neglected in workshops and seminars for lecturers because a standard level of TK is required to enable lecturers to provide an engaging online environment for students (Anderson et al., 2013; Fabian et al., 2019). In a quantitative study before the COVID-19 pandemic, Fabian et al. (2019) explored 112 lecturers’ knowledge from different disciplines regarding PCK, TK, and technological curricular-content knowledge (a subscale that combined TCK and TPK). They reported that lecturers perceived that their knowledge was lowest on TK and highest on PCK.

It is important to highlight that TPACK was designed in the context where learning how technology could be integrated with teaching was done on a voluntary basis and gradually, rather than enforced by the COVID-19 restriction measures. Furthermore, it was developed in the context where the educational environment was mainly based on face-to-face teaching rather than completely online, where technology is a starting point to access the teaching. However, the TPACK framework has been used since the COVID-19 pandemic in a number of studies to explore teachers’ preparedness to integrate technology in teaching in schools (e.g., Fuad et al., 2020) and universities (e.g., Li et al., 2021; Scherer et al., 2021); however, our literature search indicated that those studies were not conducted in the context of tertiary mathematics. For instance, in a quantitative study that was conducted in China, Li et al. (2021) explored lecturers’ perceptions about their knowledge using Likert scale items in terms of the seven components of the TPACK framework. They reported that the lecturers’ perceived knowledge was higher in the components without technology (i.e., CK, PK, PCK) compared to those that are technology related (i.e., TK, TPK, TCK, and TPACK).

We conclude this section by highlighting that the TPACK framework could capture the teaching of mathematics at the university level during the pandemic as engaging with content centred technologies such as Desmos, GeoGebra, and MATLAB could fall more under TCK and TPACK. Furthermore, using general technological tools such as Zoom and Piazza could be more related to TPK in terms of how lecturers communicate with students and also how to facilitate communications between students, for example, by using breakout rooms in Zoom to give students the opportunity to discuss the topic with their peers in small groups. This framework could also address issues related to teaching and learning of mathematics that are not technology related under CK, PK, and PCK, for example, the importance of formative assessment or building relationships with students in face to face teaching and traditional classroom with no particular use of advanced technology.

## Teaching and Learning of Mathematics in Online Settings

In the past few decades, the online medium has created new opportunities for teaching and learning across many disciplines, including mathematics. Internet development and its accessibility have dramatically changed how two-way communication could happen between students, and between students and teachers (Engelbrecht et al., 2020). New technologies have extended the concepts of classroom and lecture, and it is not easy to distinguish between inside and outside the classroom, and study and leisure time (Borba et al., 2017). Borba et al. (2017) further highlighted:

…The regular classroom no longer serves as locus for education. Couches, chairs, tables at students’ houses and cafés are the “new classrooms”. Flipped classrooms change the notion of what is in and outside of the classroom and also change the roles of students and teachers (p. 230).

Additionally, educators are facing the new generations of students who are growing up in the digital world, where computers, the internet, and online social media play a significant part in their daily lives and experiences (Engelbrecht et al., 2020).

Prior to the COVID-19 pandemic, teaching and learning of mathematics had been influenced by how different technologies (e.g., Donnelly-Hermosillo et al., 2020) and the internet (e.g., Engelbrecht & Harding, 2005) could be used for teaching and learning of mathematics. Computer-based technology was increasingly integrated into many mathematics subjects (e.g., using MATLAB to teach linear algebra (Caglayan, 2018)). Mathematics textbooks incorporated online components (see, for instance, Thomas’ calculus (Hass et al., 2018)); many universities used online homework submission and evaluation resources (e.g., Webwork^2^) and learning management systems such as Canvas^3^ were used to mediate online content delivery and student responses. Several Massive Open Online Courses (MOOCs) were designed and implemented for teaching undergraduate mathematics courses (e.g., calculus and differential equations courses) such as those offered by Coursera^4^, and blended learning approaches such as flipped classrooms were introduced in undergraduate mathematical courses. Mathematics educators have also started inquiring in this regard, exploring how students and teachers experienced engaging with MOOCs (e.g., Townsley, 2016) and flipped classrooms (e.g., Maciejewski, 2016; Naccarato & Karakok, 2015). Mobile technologies (e.g., smartphones) were also introduced as a means for teaching and learning of mathematics (see Borba et al., 2017), and several studies explored its potential (e.g., Wijers et al., 2010) and how students and teachers perceived these technologies (e.g., Holubz, 2015). Additionally, online platforms for assessment (e.g., STACK^5^) offer fresh opportunities for mathematical learning, such as giving immediate personal feedback to students and saving lecturers and students’ time (Rasila et al., 2015).

With the help of new technologies, symbolic, graphical, and interactive content can now be included in online mathematical courses (Galligan et al., 2010) in line with Bruner (1966) three modes of representation (or thinking). Animated figures and mathematical representations were found beneficial for improving students’ mathematical understanding and solving mathematical problems (Voskoglou, 2019). With the help of tablets with freehand writing tools and authoring tools/apps that permit online sharing, mathematical concepts, symbols, and solution process steps can now be communicated easily in online mathematical courses, positively impacting interaction and students’ participation in online mathematical courses (Karal et al., 2015). This technology has also been beneficial in changing some of the lectures’ negative attitudes towards online mathematics courses. They felt more comfortable teaching mathematics in online environments after using this technology (Karal et al., 2015).

Socio-cultural (e.g., the theory of commognition (Sfard, 2008)) and to some extent some of the constructivist theories toward learning (e.g., radical and social constructivism (see von Glasersfeld, 1995; Steffe & Thompson, 2000; Thompson, 2014)) highlight the importance of collaboration, sharing knowledge and understanding, and discourse for learning mathematics. Furthermore, the importance of active learning and providing opportunities for students to engage actively with learning materials and co-create knowledge is highlighted by learning theories such as the ICAP (Interactive, Constructive, Active, and Passive) framework (Chi & Wylie, 2014). Recent technologies have provided the necessary tools (e.g., discussion forums) for interactions and collaboration in online education, and mathematics educators (e.g., Engelbrecht et al., 2020; Petty & Farinde, 2013) pointed out the importance of student engagement with these tools. For instance, Petty and Farinde (2013) highlighted the importance of student engagement in the discussion forums to develop a meaningful understanding of mathematics: “If student engagement is absent or minimised, then full cognitive development in a specific content area is unachievable” (p. 263). More recently, Taranto et al. (2020) highlighted the importance of collaboration between learners in the design of MOOCs in mathematics education. They claimed that their careful design of a MOOC for mathematics teacher education resulted a higher completion rate (between 36 to 42%) compared to the average rate in the world that is 12%.

Despite the advantages of new technologies and the opportunities they have created for teaching and learning mathematics, some concerns and questions have been reported. For instance, twenty years ago, Hopper (2001) questioned if the online context could provide a nurturing environment for students and highlighted even in a traditional teaching situation, “there is a recognition of being physically present, of mutual awareness, and the student who merely listens attentively may in fact experience a highly intimate and satisfying learning and social transaction” (p. 41). About fifteen years ago, Engelbrecht and Harding (2005) raised an important question of “does the *story* of math still come across when classes move online?” (p. 255). Additionally, they highlighted the importance of a good balance between lecturer and student-centred activities in online education, and thinking about ways in which students could interact with content, lecturer, and peers (Engelbrecht & Harding, 2005). More recently, Borba et al. (2017) pointed out that online mathematics learning resources challenged the traditional image of the flow of mathematical knowledge from teachers to students, and as mathematics educators, we need to think about whether these resources are designed to foster a meaningful understanding of mathematics. Many students have now the opportunity of using these resources before using textbooks or consulting their mathematics teachers and lecturers.

Previous studies (e.g., Trenholm & Peschke, 2020; Wallace, 2003) also reported that mathematics lecturers found it challenging to design an efficient and effective online learning environment for students due to the nature of mathematics learning and the sophistication of the new technologies. In line with these findings, previous studies suggested that fully online (FO) mathematical courses were less successful than non-mathematical courses (e.g., English courses (Xu & Jaggars, 2011)). The need for having good technological knowledge and lack of face-to-face contact were found as other challenges of online teaching compared to regular teaching in which both teacher and students are physically present (e.g., Engelbrecht & Harding, 2005; Ng, 2001; Wallace, 2003). When designing web-based courses, the social nature of learning should not be overlooked and replicating the traditional teaching approaches should be avoided (Stiles, 2000). Recently, Trenholm et al. (2019) conducted a review of FO undergraduate mathematics teaching between 2000 to 2015 and concluded that FO mathematics teaching was not successful compared to traditional face-to-face teaching. For instance, Vilardi and Rice (2014) reported a significant difference in students’ mathematical achievement based on course grade point average between traditional teaching and technology-assisted course delivery methods, with students in traditional teaching outperformed their counterparts. Trenholm et al. (2019) noted that FO education in mathematics is still in the developmental phase, and challenges are expected to appear along the way. They suggested using technology-enabled peer assessment processes to increase student–student interaction, engage them in higher-level mathematical activities, and shift the teaching to a more student-centred approach.

More broadly, in educational research, there is also some evidence regarding the association between socioeconomic status and university students’ experiences of technologies (McKenzie et al., 2014). For instance, McKenzie et al. (2014) highlighted that “there may be a subtle digital divide, where financially disadvantaged students are engaging less with technologies that will most benefit their future employment” (p. 688). Furthermore, while MOOCs are perceived as an opportunity to give (free) access to individuals who are interested in higher education as a means for democratizing education, recent research suggested MOOCs need to be adapted to address better the needs of financially disadvantaged students (Dillahunt et al., 2014).

Given the findings from research noted above, and further the awareness of the possible limitations or constraints of FO mathematics teaching, we wanted to explore what happened when FO teaching was suddenly demanded of teachers and students at a national level. The lockdown imposed in reaction to the COVID-19 pandemic provides an opportunity for such exploration. The online and technology rich infrastructure within Norwegian higher education offers an ideal context for this because any limitations and constraints are likely to emerge from teachers’ and students’ competencies and knowledge, rather than technological constraint. However, we acknowledge that this is not the case everywhere, and many places do suffer from a lack of such resources.

## Methodology

We report from an exploratory sequential mixed methods study (Creswell, 2014). It started with a qualitative phase (conducting interviews with lectures and undergraduate students), and the knowledge gained in this phase was used to develop a questionnaire for a quantitative phase. The design of the instrument is a crucial element of the study because it was believed to be important to construct this to be adapted to respondents’ possible experiences rather than researchers’ preconceived ideas. In the qualitative phase, we interviewed ten mathematics lecturers with varying lengths of teaching experience in higher education and six undergraduate students. The informants responded to several open-ended questions to describe their experience of transitioning to online teaching and learning of mathematics in Norwegian higher education institutions (HEI) in 2020. These questions emerged out of several discussions between the authors. The interviews were conducted online (using Zoom) and recorded. One researcher conducted the interview, a second listened in and made notes; only at the end of the interview did the second researcher enter the conversation and explore some of the issues that had arisen in greater depth.

The responses from the interviews were used as the basis for developing the questionnaire. The first draft of the questionnaire was designed in English and sent to several well-known tertiary mathematics education researchers. Their feedback was used to make some improvements to the structure of the questionnaire; however, the intention and content of the items remained unchanged. After this refinement, the questionnaire was translated into Norwegian, and an experienced Norwegian mathematics lecturer checked the translation. Given the careful process followed for the construction of the instrument, we claim that the breadth of issues addressed by the instrument is greater than would be the case if the instrument were based only on the researchers’ thought experiment. Using convenience sampling, we distributed the instrument (Norwegian and English versions) via an online survey tool (SurveyXact) to mathematics lecturers in Norwegian HIE through the MatRIC^6^ Contact Group. The stages of instrument preparation, distribution data collection and analysis were approved in advance by the Norwegian data protection agency (NSD^7^). NSD considers both the General Data Protection Regulations (GDPR) and ethical issues arising from the collection of personal data.

### Study Participants

One hundred and twenty-seven students and eighteen lecturers completed the survey^8^. The students were from four Norwegian universities; however, the majority (90%) participated were from one university. If we add the students interviewed earlier regarding online teaching and learning of mathematics in the lockdown period, there are representatives from five universities altogether. This is insufficient to make any claims about representativeness, but it may be sufficient to validate some of the key responses from the lecturers.

Regarding the lecturer sample group, seven universities are represented in the responses to the online survey; there were additional institutions represented in the interviews. In total, ten institutions are represented, including Norway’s major universities. As with the students, there were relatively few lecturers who completed the survey. In total, 18 individual respondents plus up to ten additional respondents from the interviews (assuming the interviewees did not also complete the survey), it is not likely to be genuinely representative of the Norwegian HEI mathematics lecturing community. However, the fact that the largest institutions are represented will enable insight into institutional characteristics, and the respondents will give a sense of variations within and across institutions.

The lecturers who participated in the survey had a wide range of experiences of teaching mathematics in higher education as a lecturer/associate professor/professor from a minimum of 3 to a maximum of 39 years. The mean of their teaching experience was 14.07 years, with a standard deviation of 11.28. Regarding the student sample group (Table 1), the majority (90%) were first and second-year students. Therefore, care should be taken in interpreting the findings due to the skewed distribution.

**Table 1:** Students’ study year

Study year	N	%
First-year	86	67.7
Second-year	28	22.0
Third-year	8	6.3
Fourth-year or higher	5	3.9

The gender distribution of the participants is provided in Table 2, indicating the student sample group was well-balanced with respect to gender. However, that was not the case for the lecturer cohort.

**Table 2:** The gender distribution of the participants

	Male		Female		Missing information	
	N	%	N	%	N	%
Students	59	46.5	57	44.9	11	8.7
Lecturers	15	83.3	2	11.1	1	5.6

### Instrument

The refined instrument had several sections. It started with exploring background information about the participants. Then several questions were related to teaching and learning practices before the lockdown. The remaining parts were dedicated to teaching and learning mathematics in online settings, such as communication between the lecturer and individual students, challenges of learning and teaching mathematics online, assessment in an online setting, and participants’ perceptions of the psychological impact of lockdown. In the next section, we share the main study findings.

## Results

We present the findings in three sections. We start by describing participants’ prior experience of online education and then present the challenges they experienced during online education. We finish the results section by describing the psychological impact of lockdown on students and lecturers.

### Prior Experience of Online Teaching and Learning of Mathematics

Lecturers and students were asked about their experience of online teaching/learning before the lockdown. The responses (Table 3) show that students had more experience of engaging with online education than the lecturers as 71.2% of students selected the first three items, whereas this percentage was only 38.9% for lecturers. Additionally, one can observe that 16.7% of lecturers had no online education experience before the lockdown, and 44.4% of them *chose poor, little familiarity, limited use*; however, these percentages for students were 4.7% and 23.6%, respectively. We also used Fisher’s exact test to examine the association between the responses of students and lecturers to the items of the questionnaire. The findings indicate that there was a significant difference (*p* = 0.072^9^) between the experience of students and lecturers regarding online education before the lockdown. One needs to be very careful about drawing direct comparisons between lecturers and students. It appears in the responses to the questionnaire, a much higher proportion of students had some prior experience of using online learning resources than the proportion of lecturers that had prior to using online or digital resources in their teaching. This could impact later responses to questions because the cohort of students responding had greater familiarity with online teaching/learning than the lecturers.

**Table 3:** The experience of students and lecturers regarding online education before the lockdown

Items	Students		Lecturers	
	N	%	N	%
1. Extensive, very experienced	7	5.5	0	0
2. Good, familiar with most programs/Apps and used some of them	35	27.6	3	16.7
3. Moderate, familiar with some programs/Apps but little experience of use	49	38.6	4	22.2
4. Poor, little familiarity, limited use	30	23.6	8	44.4
5. Non existent	6	4.7	3	16.7
Fisher’s Exact Test	0.072			

### Challenges of Learning and Teaching Mathematics Online

Twelve items (Table 4) were designed to explore the challenges of online learning and teaching mathematics. These items were derived from the interviews that were conducted in the first phase of the study. The informants had six choices for each item, and they were different for lecturers and students, as shown in Table 4. The p-values corresponding to these items indicate a significant difference between how lecturers and students responded to these items. One possible reason for such differences is that the choices were different.

**Table 4:** Challenges students experienced during the lockdown period of online teaching

Student choices	Did not experience at all		I experienced some challenge but I took effective action and did not suffer		I experienced some challenge and took some action to get help, but the challenge remained		Experienced this moderately		Experienced very much		Not applicable		Fisher’s Exact Test
Lecturer choices	Did not consider this as important or relevant		Considered and felt it was students’ responsibility to take action		Considered and took some action		Considered deeply and took moderate action		Considered deeply and took strong action		Not applicable		
	N	%	N	%	N	%	N	%	N	%	N	%	
S1. Social isolation and missing friends and colleagues to work with	12	9.8	28	22.8	14	11.4	32	26	35	28.5	2	1.6	Less than
L1. Students would suffer social isolation and miss friends and colleagues to work with	1	5.6	6	33.3	6	33.3	2	11.1			3	16.7	0.001
S2. Missing the routine and structure of coming to university and following the daily schedule	14	11.4	20	16.3	9	7.3	23	18.7	55	44.7	2	1.6	Less than
L2. Students would miss the routine and structure of coming to university and following the daily schedule			6	33.3	4	22.2	2	11.1	2	11.1	4	22.2	0.001
S3. Missing the availability or physical presence of the lecturer or student learning assistants to ask questions	19	15.4	22	17.9	10	8.1	32	26	39	31.7	1	.8	Less than
L3. Students would miss the availability or physical presence of the lecturer or student learning assistants to ask questions			1	5.6	11	61.1	2	11.1	1	5.6	3	16.7	0.001
S4. Missing the live presentation of mathematics by a physically present lecturer	17	13.8	26	21.2	8	6.5	23	18.7	44	35.8	5	4.1	Less than
L4. Students would miss the live presentation of mathematics by a physically present lecturer	2	11.1			7	38.9	3	16.7	2	11.1	4	22.2	0.001
S5. Too many distractions at home (by other people, pets, entertainment, etc.)	19	15.4	28	22.8	19	15.4	25	20.3	28	22.8	4	3.3	0.001
L5. Students would be distracted at home (by other people, pets, entertainment, etc.)	1	5.6	10	55.6	3	16.7	1	5.6			3	16.7	
S6. Increased anxiety because there were no other students to help pace the progress through the mathematics or to measure progress against	39	31.7	20	16.3	15	12.2	27	22	16	13	6	4.9	0.006
L6. Students would experience increased anxiety because they have no other students to help them pace themselves or measure their own progress against	3	16.7	6	33.3	4	22.2	1	5.6			4	22.2	
S7. Lacking necessary or adequate resources (broadband, computer, writing tablet, etc.)	91	71.4	14	11.4	6	4.9	5	4.1	3	2.4	4	3.3	Less than
L7. Students would not possess necessary or adequate resources (broadband, computer, writing tablet, etc.)	5	27.8	6	33.3	2	11.1					5	27.8	0.001
S8. Discomfort with the loss of anonymity or privacy in using social media chat forums for sharing mathematical difficulties	73	59.3	11	8.9	3	2.4	11	8.9	10	8.1	15	12.2	0.002
L8. Students would be uncomfortable with the loss of anonymity or privacy in using social media chat forums for sharing their mathematical difficulties	4	22.2	4	22.2	4	22.2	2	11.1	1	5.6	3	16.7	
S9. Lacking motivation or confidence to come online to ask questions and get help	29	23.6	25	20.3	11	8.9	34	27.6	20	16.3	4	3.3	Less than
L9. Students would lack motivation or confidence to come online to ask questions and get help	2	11.1	4	22.2	8	44.4	1	5.6			3	16.7	0.001
S10. Difficult to complete assignments, especially assignments based on group activity	22	17.9	23	18.7	12	9.8	31	25.2	18	14.6	17	13.8	0.05
L10. Students would experience difficulty in completing assignments, especially assignments based on group activity	2	11.1	4	22.2	5	27.8	2	11.1			5	27.8	
S11. The requirement to take more responsibility for my own learning	30	24.4	28	22.8	13	10.6	29	23.6	20	16.3	3	2.4	Less than
L11. Students would need to take more responsibility for their own learning			4	22.2	11	61.1					3	16.7	0.001
S12. The shock experienced when the lockdown was suddenly imposed and finding it difficult to adapt to the new teaching/learning routine	33	26.8	31	25.2	12	9.8	26	21.2	18	14.6	3	2.4	Less than
L12. Students would experience a shock and find it difficult to adapt themselves to the new teaching/learning routine	2	11.1	2	11.1	8	44.4	1	5.6			5	27.8	0.001

The challenges could be categorised into three groups based on how much students experienced them. The first group consisted of five challenges (Items 1 to 5: social isolation, loss of routine, missing physical presence of lecturer or students, missing live presentation, distractions), and they were experienced by more than 80% of students (i.e., 81.3% to 88.6%). The second group also consisted of five challenges (Items 6: missing other students against whom to judge pace; and 9 to 12: lacking motivation or confidence to seek help, completing assignments, greater responsibility for self, shock and difficulty to adapt); however, they were experienced less than the first group (63.4% to 73.2%). Finally, the third group of challenges (Items 7 & 8: lacking resources, loss of anonymity) was experienced by less than 30% of students (26.2% to 28.5%). Additionally, across these twelve challenges, we can observe that for ten of them, one in every three students experienced them *moderately* or *very much* during the lockdown. More importantly, more than half the students experienced four of the challenges: *social isolation and missing friends and colleagues to work with (Item 1)*; *missing the routine and structure of coming to university and following the daily schedule (Item 2)*; *missing the availability or physical presence of the lecturer or student learning assistants to ask questions (Item 3)*; and *missing the live presentation of mathematics by a physically present lecturer (Item 4)*. We begin with taking a closer look at the first group in the following paragraphs.

Regarding *social isolation and missing friends and colleagues to work with*, around 90% of students experienced this challenge. More importantly, almost 55% of them experienced it *moderately* or *very much*. However, looking at the lectures’ responses, around 55% of them chose *did not consider this as important or relevant*, *considered and felt it was students’ responsibility to take action,* or *not applicable*. The second challenge, *missing the routine and structure of coming to university and following the daily schedule*, was also experienced by many students (i.e., 87%). Among them, 63.4% experienced it *moderately* or *very much.* The lecturers’ responses show that 55.5% of them chose *considered and felt it was students’ responsibility to take action* or *not applicable.* These findings suggest that mathematics lecturers would benefit from raising their awareness about the challenges students experienced during online education.

*Missing the availability or physical presence of the lecturer or student learning assistants to ask questions* were experienced by 83.8% of students. Among these students, 57.7% experienced this challenge *moderately* or *very much*. This challenge was recognised much more by the lecturers than the previous two challenges as only 22.2% of them chose *considered and felt it was students’ responsibility to take action* or *not applicable*. The remaining lecturers considered this challenge and took *some*, *moderate* or *strong action* to help students overcome it. Regarding *missing the live presentation of mathematics by a physically present lecturer*, similar responses were observed; 82.1% of students experienced this challenge. Among them, 54.5% experienced this challenge *moderately* or *very much*. Looking at the lectures’ responses, two-third of them perceived that they considered this challenge and took *some*, *moderate*, and *strong action* to help students with this challenge.

For *too many distractions at home (by other people, pets, entertainment, *etc*.)*, 81.3% of students experienced this challenge. Among them, 43.1% experienced it *moderately* or *very much*. Lecturers’ responses indicate that only 22.3% perceived this challenge and took *some* or *moderate action* toward it to help students overcome this challenge. One could argue that it is hard to know what actions a lecturer could take to alleviate the situation for students and therefore some lecturers took a defensive accommodation toward this item.

For the second group of challenges, 35% of students *moderately* or *very much* experienced *increased anxiety because there were no other students to help pace the progress through the mathematics or measure progress against*. However, only 27.8% of lecturers perceived that they considered it and took *some* or *moderate* actions. Regarding *lacking motivation or confidence to come online to ask questions and get help*, 43.9% of students *moderately* or *very much* experienced it, whereas 50% of lecturers perceived that they considered it and took *some* or *moderate* action. 39.8% of students *moderately* or *very much* experienced difficulty with completing assignments, especially assignments based on group activity. A similar percentage of lecturers (i.e., 38.9%) perceived that they considered it and took *some* or *moderate* action toward this challenge. The requirement to take more responsibility for own learning was *moderately* or *very much* experienced by 39.9% of students. 61.1% of the lecturers perceived that they considered it and took some action. Regarding item 12, *the shock experienced when the lockdown was suddenly imposed and finding it difficult to adapt to the new teaching/learning routine*, 35.8% of students *moderately or very much* experienced this challenge while 50% of lecturers perceived that they considered it and took *some* or *moderate* action towards it.

The third group of challenges markedly affected fewer students, less than 20% of students *moderately* or *very much* experienced *discomfort with the loss of anonymity or privacy in using social media chat forums for sharing mathematical difficulties*. A lower percentage of students (less than 10%) *moderately* or *very much* experienced *lacking necessary or adequate resources (broadband, computer, writing tablet, *etc*.).* Similarly, 61.1% and 88.9% of lecturers chose *did not consider this as important or relevant*, *considered and felt it was students’ responsibility to take action*, or *not applicable*.

To summarise, one could conclude that many of the challenges students experienced were not anticipated or considered by the lecturers to be part of their responsibility. A piece of evidence supporting this claim is that across the twelve challenges listed in the lecturer questionnaire, for nine of them, at least 50% of the lecturers selected one of the following three choices: *did not consider this as important or relevant*, *considered and felt it was students’ responsibility to take action*, and *not applicable*.

After responding to these twelve items, the participants were invited to describe other challenges students experienced during the lockdown. Six main challenges were identified from the responses: difficulty with children and other family members; lacking or losing motivation; group or partner collaboration did not function well; difficulty in getting mathematics help when wanted/needed; inadequate information about the subject, or the lecturer not well-adjusted to the changed circumstances, competition with other subjects’ demands; and losing contact with university health support.

We also explored the possible gender difference between the challenges students experienced during the lockdown as our sample had a good balance between male and female students (see Table 2). We used Fisher’s Exact test to explore the association (Appendix A) and conducted the post hoc multiple Z-test to have pairwise comparisons among the six choices, and we adjusted the p-values using the Bonferroni correction in SPSS. As can be seen in Appendix A, three significant differences existed between the responses of male and female students regarding Item 1, 7, and 10. The post hoc analysis regarding the first item, *social isolation and missing friends and colleagues to work with*, indicates that the proportion of male students (18.6%) who chose *I experienced some challenge and took some action to get help, but the challenge remained* was significantly higher compared to female counterparts (3.5%). For Item 7 (i.e., *lacking necessary or adequate resources *(*broadband, computer, writing tablet, *etc*.*)), a higher proportion of male students (84.7%) selected *did not experience at all* than female students (64.9%). Additionally, a higher proportion of female students (8.8%) selected *experienced this moderately* compared to male students (0%). Finally, regarding Item 10, *difficult to complete assignments, especially assignments based on group activity*, a higher portion of male students (27.1%) chose *did not experience at all* than female students (7%).

We also invited the lecturers to describe the challenges they experienced during the lockdown and online education by responding to 12 items listed in Table 5. These items were also derived from the interviews. Looking at the percentages of lecturers who chose *moderately challenging* or *very challenging,* we can observe that the highest challenge the lecturers experienced was related to *monitoring students’ understanding and learning,* with 94.5% of the lecturers selecting one of these choices. Additionally, we can observe that the second and third highest challenges were *meeting student’s individual* needs and *getting feedback from students about whether they are understanding* mentioned by 83.3% and 77.8% of lecturers, respectively. Within the remaining nine challenges, four of them were perceived *moderately* or *very challenging* by at least 50% of lecturers during the lockdown. These challenges were: *giving students feedback about their learning and progress* (61.1%); *additional work involved in moving to online provision* (61.1%); *the same writing surface could not be shared by the students and me simultaneously while writing* (55.5%); and *motivating students to engage with the mathematics* (50%).

**Table 5:** Lecturers’ challenges during the lockdown and online teaching

Items	Not a big problem		Low level of challenge		Moderately challenging		Very challenging		Neutral, not my responsibility		Not relevant	
	N	%	N	%	N	%	N	%	N	%	N	%
1. Motivating students to engage with the mathematics	4	22.2	4	22.2	6	33.3	3	16.7			1	5.6
2. Encouraging students to reduce their anxiety and stress	9	50	4	22.2	2	11.1	1	5.6	2	11.1		
3. Meeting student’s individual needs	2	11.1	2	11.1	9	50	5	27.8				
4. Getting feedback from students about whether they are understanding			3	16.7	7	38.9	8	44.4				
5. Monitoring students' understanding and learning			1	5.6	7	38.9	10	55.6				
6. Giving students feedback about their learning and progress	2	11.1	5	27.8	8	44.4	3	16.7				
7. Producing lectures or mini-lectures from "home-office"	11	61.1	4	22.2			2	11.1			1	5.6
8. Streaming lectures or taking part in online sessions from home (maybe because of inadequate bandwidth or competition with other members of the household for broadband connection)	6	33.3	3	16.7	2	11.1	2	11.1			5	27.8
9. The same writing surface could not be shared by the students and me simultaneously while writing	1	5.6	2	11.1	6	33.3	4	22.2			5	27.8
10. Writing and posting online mathematical text	12	66.7	4	22.2	1	5.6	1	5.6				
11. Additional work involved in moving to online provision	2	11.1	5	27.8	9	50	2	11.1				
12. Encouraging and facilitating students to work in groups	5	27.8	1	5.6	4	22.2	3	16.7	3	16.7	2	11.1

### The Psychological impact of Lockdown on Students and Lecturers

This section explores the psychological impact of the lockdown on the students and lecturers (Table 6). The participants were asked to indicate how the issues set out in items in Table 6 affected learning and teaching during the lockdown*.* Item 7 (relating to personally experiencing financial problems) was not included in the lecturer questionnaire as we believed this would not be relevant to lecturers in the Norwegian context. The findings indicate two significant differences in the responses of students and lecturers in the following items: *Fear for myself and/or my family of being infected by the virus* and *uncertainty about the future for myself and/or my family.* In response to both sources of possible anxiety, it seems students’ learning was more affected than lecturers’ teaching. Additionally, the descriptive statistics presented in Table 6 show that universities and lecturers need to be mindful of the well-being of their students and how that impacts their learning. Between 20 to 42% of the students selected *some* or *very much* in response to these items with the highest percentage (i.e., 41.9%) for *absence from workplace and colleagues*.

**Table 6:** The psychological impact of lockdown on student learning and lecturer teaching

Items	Category	Not at all		A little		Some		Very much		I prefer not to comment		Fisher’s Exact Test
		N	%	N	%	N	%	N	%	N	%	
1. Fear for myself and/or my family of being infected by the virus	Students	36	30.8	46	39.3	20	17.1	15	12.8			.028
	Lecturers	12	6.7	5	27.8	1	5.5					
2. Losing physical contact with family and friends	Students	39	33.3	35	29.9	21	18	22	18.8			.176
	Lecturers	8	44.4	7	38.9	3	16.7					
3. Distractions of family and working at home	Students	33	28.2	39	33.3	26	22.2	16	13.7	3	2.6	.836
	Lecturers	7	38.9	6	33.3	4	22.2	1	5.6			
4. Absence from workplace and colleagues	Students	33	28.2	32	27.4	26	22.2	23	19.7	3	2.6	.278
	Lecturers	3	16.7	10	55.6	3	16.7	2	11.1			
5. Uncertainty about the future for myself and/or my family	Students	51	43.6	31	26.5	17	14.5	16	13.7	2	1.7	.089
	Lecturers	13	72.2	5	27.8							
6. Depression	Students	62	53	27	23.1	14	12	9	7.7	5	4.3	.695
	Lecturers	13	72.2	3	16.7	2	11.1					
7. Financial issues for myself and/or my family	Students	67	57.3	26	22.2	15	12.8	7	6	2	1.7	NA
	Lecturers	Not applicable (NA)										

Table 7 shows the perceptions of students and lecturers about the consequences of online teaching in the long term on student learning and outcomes as well as the prospects of the survival of smaller institutions. Fisher’s exact test shows a significant difference between the perceptions of students and lecturers in the first item. 94.4% of lecturers perceived that students’ learning experiences would get *a lot worse* or *worse* if the online teaching continues in the long term, whereas this percentage was lower for students (62.4%). Regarding the second item, similarly, 94.4% of lecturers were concerned about students’ learning outcomes, whereas that percentage was lower for students (64.1%). Looking at the descriptive statistics shared in Table 7, one might conclude that some students had a positive perception of online teaching as 21.4% of students selected *improve moderately* or *improve a lot* in responding to the first item, and 14.5% chose these two options in responding to the second item. However, no lecturers selected these two options in responding to these items.

**Table 7:** Perceptions of participants on the consequences of online teaching in the long term

Items	Category	Get a lot worse		Get worse		No change		Improve moderately		Improve a lot		No idea		Fisher’s Exact Test
		N	%	N	%	N	%	N	%	N	%	N	%	
Students’ learning experiences	Students	20	17.1	53	45.3	13	11.1	20	17.1	5	4.3	6	5.1	.099
	Lecturers	6	33.3	11	61.1							1	5.6	
Students’ learning outcomes	Students	25	21.4	50	42.7	20	17.1	13	11.1	4	3.4	5	4.3	.131
	Lecturers	6	33.3	11	61.1							1	5.6	
Prospects for the survival of smaller institutions	Students	21	17.9	30	25.6	10	8.5	6	5.1	4	3.4	45	38.5	.379
	Lecturers	1	5.6	4	22.2	2	11.1	3	16.7			8	44.4	

We also explored gender difference in the psychological impact of the lockdown on the students (Appendix B) using the same approach described above for investigating gender differences in challenges students experienced. Two significant differences were found for Item 4 (absence from the workplace) and 7 (financial issues) at 0.10 level. Regarding *absence from workplace and colleagues*, the post hoc analysis, as work at 0.05 level, does not identify any significant difference between male and female responses to the choices for this item. However, for Item 7, *financial issues for myself and/or my family*, a lower portion of male students (11.9%) selected *a little* compared to female counterparts (33.3%). Finally, no significant difference was found (Appendix C) between male and female students regarding their perceptions of the consequences of online teaching in the long term.

## Discussion and Conclusion

In this exploratory sequential mixed study, we reported on the perceived experiences of mathematics lecturers and university students in Norway when they transitioned to fully online education in 2020 due to the COVID-19 pandemic. We hope the findings presented here could increase the awareness of mathematics lecturers and tertiary mathematics educators about the challenges mathematics lecturers and students experienced during fully online teaching and learning mathematics, and they work together with the help of university decision-makers and administrative staff to address these challenges as similar lockdown restrictions, and fully online education might be experienced in the (near) future. If online education is going to become a significant part of tertiary mathematics education, it would be necessary to work out responsibilities, structures, and strategies to address the challenges students and lecturers experience.

Several points can be taken from the results shared in this paper regarding the challenges of teaching and learning mathematics online. Consistent with Trenholm et al. (2019) study, the findings suggest that online education for tertiary mathematics is still in the developmental phase and many students and lecturers face challenges when working in such an environment. Advanced technologies and the internet, at least in the context of teaching and learning of mathematics, could not yet replace the experience students have in face-to-face teaching and the collaborative learning environments at the universities. The issue of lack of interactions and collaboration in online education were highlighted in studies between 2000 and 2005 (i.e., Engelbrecht & Harding, 2005; Ng, 2001; Wallace, 2003) but, more than 15 years later, the new generation of students still experience those issues and the advanced technology and the accessibility of internet have not yet resolved them. It seems the points Ng highlighted in 2001 are still relevant: A new set of social and communication skills are needed for online education, and it seems some students do not feel comfortable communicating with their fellow students that they do not know from the past. Students have become accustomed to social media in which anything they write or post can be visible to all, the online environment lacks the privacy and intimacy that students may prefer when revealing their uncertainties in a learning situation. In other words, they fear being exposed to online course/learning “trolls”. Additionally, students’ anxiety could increase in online communication when waiting for a reply from others or when communicating with text-based tools such as email where non-verbal and oral cues are absent.

Many universities in Norway and internationally require the new permanent staff to participate in a university pedagogy programme. These programmes typically help lecturers to develop their PK and also reflect on their PCK in the context of higher education. How technology could be integrated into teaching and learning and how technology could facilitate or hinder learning are discussed in some of the modules in such programmes that help lecturers develop their TK and TPK. Furthermore, the participants are usually encouraged to reflect on how different technologies could be used in their subjects that could also help lecturers develop their TPACK. However, the findings suggest that more weight should be given in such programmes to how technology could be integrated into teaching and what are possible psychological challenges in online environments for students to prepare (mathematics) lecturers for the new norm and to develop their TK, TPK, and TPACK. This suggestion is supported by Li et al. (2021) findings that lecturers perceived that their technology-related knowledge is not well developed compared to their CK, PK, and PCK. In addition, it is also in line with Fabian et al. (2019) findings that the lecturers perceived their TK as less developed than their PCK.

Sharing the study findings regarding the challenges students faced during online education (Table 4) and the psychological impact of lockdown on students’ well-being (Table 6) with mathematics lecturers could help them to develop their TPK further. Disseminating the findings with decision-makers and administrative staff at the tertiary level could also help with planning for future similar situations. It is important to communicate with them that many students experienced social isolation, increased anxiety, fear, distraction, missing routines and physical presence, lacking motivation to ask questions, feeling uncertainty during the lockdown and online education, and difficulty in adapting to the new environments that all could negatively impact their learning. We should highlight that lecturers with awareness and sensitivity to these issues can make a positive impact to help students adapt to the new environment.

The findings set out in Table 3 suggest some aspects of lecturers’ TK and TPK were less developed than university students before the lockdown. This is not a surprising finding considering the new generation of students have grown up with technology and the internet (Engelbrecht et al., 2020) and these are integrated into their daily lives much more compared to their mathematics lecturers that are from older generations where technology and the internet were not that much developed. Additionally, the findings provided in Table 5 indicate the degree to which lecturers experienced challenges related to their TPK and TPACK. For instance, many lecturers struggled to meet students’ individual needs and monitoring their understanding and learning. Furthermore, the results presented in Table 4 indicate that some of the lecturers were not aware of some of the challenges students experienced during the lockdown. For example, as highlighted in the results section, social isolation and missing friends and colleagues to work with were overlooked by several lecturers. Running workshops and seminars for disseminating these findings, sharing successful ways in which technology and the internet could be integrated with online teaching and learning, preferably in mathematics, might be more crucial in the current situation to help mathematics lecturers develop their TK, TPK, and more importantly, their TPACK.

The final point before discussing the limitations is highlighting and acknowledging the existing literature in mathematics education both in school (e.g., Donnelly-Hermosillo et al., 2020) and university (e.g., Maciejewski, 2016) that focuses on integrating technology in teaching and learning, and more importantly, the literature on teaching and learning of mathematics in online settings (e.g., Townsley, 2016). Research in both school and tertiary mathematics education has a growing body of literature that can assist mathematics lecturers in moving forward and how to address students’ challenges. We live in an era where many applications can be used for representing and discussing mathematical concepts. Many online platforms also ease the communication between students and lecturers and within students. For instance, mathematics lecturers could learn from the design of successful MOOCs (e.g., Taranto et al., 2020) in terms of what type of resources or tasks are useful for students and also how to facilitate peer-to-peer interaction in an online environment as learning with MOOCs is to some extent similar to learning during the lockdown.

This study has a number of limitations. First, the sample size for the quantitative phase was not large, and therefore, care should be taken in interpreting the study findings. Secondly, the items (Table 4) developed to explore the challenges students experienced during the lockdown and online education focused more on the technological aspect of lecturers’ knowledge (i.e., TK, TPK, and TPACK), and therefore, this study has not explored the challenges students experienced regarding lecturers’ CK, PK, and PCK, and therefore further research is needed to examine the challenges students experienced regarding these three knowledge types. On the other hand, we could argue that the findings of the interviews did not suggest that students’ challenges with learning mathematics during the COVID-19 pandemic are mainly related to lecturers’ CK, and therefore we did not have a closed-ended item regarding this aspect of lecturers’ knowledge in the questionnaire^10^. This could be because mathematics lecturers in Norway typically have a PhD in mathematics or mathematics education and therefore are equipped with the necessary CK for teaching the courses. Considering the majority of student participants were first or second-year students, one could argue that these courses are not mathematical demanding for lecturers, and if the sample were skewed towards postgraduate students, the findings might be different. In addition, the relative lack of privacy in an online setting is an important contrast with in-person learning, and further research could explore more deeply how this matter hinders or constrains students’ learning.

## Appendix A

### Gender Difference in Challenges Students Experienced

**Table:**

	Did not experience at all				I experienced some challenge but I took effective action and did not suffer				I experienced some challenge and took some action to get help, but the challenge remained				Experienced this moderately				Experienced very much				Not applicable				Fisher’s Exact Test
Items	Male		Female		Male		Female		Male		Female		Male		Female		Male		Female		Male		Female		
	N	%	N	%	N	%	N	%	N	%	N	%	N	%	N	%	N	%	N	%	N	%	N	%	
S1	4	6.8	8	14	15	25.4	12	21.2	11	18.6^*^	2	3.5	11	18.6	17	29.8	16	27.1	18	31.6	2	3.4			.041
S2	5	8.5	9	15.8	8	13.6	10	17.5	6	10.2	1	1.8	10	16.9	12	21.1	28	47.5	25	43.9	2	3.4			.220
S3	10	16.9	9	15.8	13	22	8	14	5	8.5	5	8.8	15	25.4	12	21.1	15	25.4	23	40.4	1	1.7			.526
S4	9	15.3	6	10.5	14	23.7	11	19.3	5	8.5	3	5.3	11	18.6	9	15.8	18	30.5	25	43.9	2	3.4	3	5.3	.718
S5	9	15.3	10	17.5	12	20.3	14	24.6	11	18.6	8	14	10	16.9	12	21.1	14	23.7	13	22.8	3	5.1			.643
S6	20	33.9	17	29.8	8	13.6	11	19.3	5	8.5	9	15.8	14	23.7	11	19.3	9	15.3	7	12.3	3	5.1	2	3.5	.771
S7	50	84.7^*^	37	64.9	4	6.8	9	15.8	1	1.7	3	5.3			5	8.8^*^	1	1.7	2	3.5	3	5.1	1	1.8	.028
S8	36	61	32	56.1	5	8.5	6	10.5	1	1.7	1	1.8	5	8.5	6	10.5	4	6.8	5	8.8	8	13.6	7	12.3	.982
S9	16	27.1	13	22.8	15	25.4	9	15.8	5	8.5	5	8.8	15	25.4	16	28.1	6	10.2	12	21.1	2	3.4	2	3.5	.574
S10	16	27.1^*^	4	7	9	15.3	14	24.6	6	10.2	5	8.8	12	20.3	18	31.6	8	13.6	9	15.8	8	13.6	7	12.3	.077
S11	17	28.8	12	21.1	12	20.3	14	24.6	8	13.6	5	8.8	13	22	13	22.8	7	11.9	12	21.1	2	3.4	1	1.8	.671
S12	20	33.9	12	21.1	10	16.9	20	35.1	5	8.5	6	10.5	14	23.7	8	14	8	13.6	10	17.5	2	3.4	1	1.8	.164

^*^The post hoc analysis showed a significant difference in the proportion of male and female students responding to this choice in the item

## Appendix B

### Gender Differences in the Psychological Impact of the Lockdown on the Students

**Table:**

	Not at all				A little				Some				Very much				I prefer not to comment				Fisher’s Exact Test
Items	Male		Female		Male		Female		Male		Female		Male		Female		Male		Female		
	N	%	N	%	N	%	N	%	N	%	N	%	N	%	N	%	N	%	N	%	
1	19	32.2	17	29.8	22	37.3	23	40.4	9	15.3	11	19.3	9	15.3	6	10.5					.836
2	22	37.3	17	29.8	15	25.4	20	35.1	10	16.9	10	17.5	12	20.3	10	17.5					.696
3	19	32.2	13	22.8	18	30.5	21	36.8	14	23.7	12	21.1	5	8.5	11	19.3	3	5.1			.165
4	12	20.3	20	35.1	19	32.2	13	22.8	16	27.1	10	17.5	9	15.3	14	24.6	3	5.1			.074
5	25	42.4	25	43.9	15	25.4	16	28.1	10	16.9	7	12.3	8	13.6	8	14	1	1.7	1	1.8	.961
6	31	52.5	30	52.6	14	23.7	13	22.8	7	11.9	7	12.3	5	8.5	4	7	2	3.4	3	5.3	1
7	38	64.4	28	49.1	7	11.9^*^	19	33.3	7	11.9	8	14	5	8.5	2	3.5	2	3.4			.028

^*^The post hoc analysis showed a significant difference in the proportion of male and female students responding to this choice in the item

## Appendix C

### Gender Differences in Students’ Perception of the Consequences of Online Teaching in the Long Term

**Table:**

	Get a lot worse				Get worse				No change				Improve moderately				Improve a lot				No idea				Fisher’s Exact Test
Items	Male		Female		Male		Female		Male		Female		Male		Female		Male		Female		Male		Female		
	N	%	N	%	N	%	N	%	N	%	N	%	N	%	N	%	N	%	N	%	N	%	N	%	
1	9	15.3	11	19.3	23	39	29	50.9	8	13.6	5	8.8	12	20.3	8	14	3	5.1	2	3.5	4	6.8	2	3.5	.688
2	12	20.3	13	22.8	21	35.6	28	49.1	13	22	7	12.3	8	13.6	5	8.8	1	1.7	3	5.3	4	6.8	1	1.8	.306
3	8	13.6	13	22.8	19	32.2	11	19.3	4	6.8	6	10.5	5	8.5	1	1.8	4	6.8	1	1.8	19	32.2	25	43.9	.119

## Footnotes

1 For instance, in Norway, according to the Norwegian Institute of public health (www.fhi.no), 1054 people died between February 2020 and November 2021 because of COVID-19, even though Norwegian authorities (and public response) were initially very successful in keeping numbers low. As of today (30^th^ of November, 2021), data from John Hopkins Coronavirus Resource Centre (coronavirus.jhu.edu) reports that the death rate in Norway is about 13% of that for Sweden and 40% of that in Denmark.

2 http://www.webwork.maa.org/

3 https://www.canvas.net/

4 https://www.coursera.org/

5 https://www.ed.ac.uk/maths/stack

6 https://www.matric.no/

7 https://www.nsd.no/en

8 A copy of the survey instrument may be obtained on request to the authors.

9 We chose p-value of less or equal 0.1 as significant because of the small sample size.

10 Students could have mentioned it in the open-ended items if they have periceved that their challenges with learning in online settting is related to lecturers’s CK.
